# Carrying asymptomatic gallstones is not associated with changes in intestinal microbiota composition and diversity but cholecystectomy with significant dysbiosis

**DOI:** 10.1038/s41598-021-86247-6

**Published:** 2021-03-23

**Authors:** Fabian Frost, Tim Kacprowski, Malte Rühlemann, Stefan Weiss, Corinna Bang, Andre Franke, Maik Pietzner, Ali A. Aghdassi, Matthias Sendler, Uwe Völker, Henry Völzke, Julia Mayerle, Frank U. Weiss, Georg Homuth, Markus M. Lerch

**Affiliations:** 1 Department of Medicine A, University Medicine Greifswald, Greifswald, Germany; 2 Division Data Science in Biomedicine, Peter L. Reichertz Institute of TU Braunschweig and Hannover Medical School, Braunschweig, Germany; 3 Institute of Clinical Molecular Biology, Christian Albrechts University of Kiel, Kiel, Germany; 4 Department of Functional Genomics, Interfaculty Institute for Genetics and Functional Genomics, University Medicine Greifswald, Greifswald, Germany; 5 Institute of Clinical Chemistry and Laboratory Medicine, University Medicine Greifswald, Greifswald, Germany; 6 Institute for Community Medicine, University Medicine Greifswald, Greifswald, Germany; 7 Department of Medicine II, University Hospital, LMU Munich, Munich, Germany

**Keywords:** Biliary tract disease, Dysbiosis

## Abstract

Gallstone disease affects up to twenty percent of the population in western countries and is a significant contributor to morbidity and health care expenditure. Intestinal microbiota have variously been implicated as either contributing to gallstone formation or to be affected by cholecystectomy. We conducted a large-scale investigation on 404 gallstone carriers, 580 individuals post-cholecystectomy and 984 healthy controls with similar distributions of age, sex, body mass index, smoking habits, and food-frequency-score. All 1968 subjects were recruited from the population-based Study-of-Health-in-Pomerania (SHIP), which includes transabdominal gallbladder ultrasound. Fecal microbiota profiles were determined by 16S rRNA gene sequencing. No significant differences in microbiota composition were detected between gallstone carriers and controls. Individuals post-cholecystectomy exhibited reduced microbiota diversity, a decrease in the potentially beneficial genus *Faecalibacterium* and an increase in the opportunistic pathogen *Escherichia/Shigella*. The absence of an association between the gut microbiota and the presence of gallbladder stones suggests that there is no intestinal microbial risk profile increasing the likelihood of gallstone formation. Cholecystectomy, on the other hand, is associated with distinct microbiota changes that have previously been implicated in unfavorable health effects and may not only contribute to gastrointestinal infection but also to the increased colon cancer risk of cholecystectomized patients.

## Introduction

Gallstone disease represents a major worldwide health burden. In western countries 10–20% of the population are gallstone carriers causing health care costs of more than five billion dollars annually in the US alone^1,2^. Important risk factors for the development of gallstones include genetic predisposition, female sex, advanced age and obesity^3^. It is expected that the ongoing global obesity epidemic will be followed by a sharp rise in gallstone prevalence. Although most affected individuals remain asymptomatic during their lifetime, 10–25% will develop symptomatic gallstone disease, the consequences of which can range from simple biliary colic to potentially life-threatening complications such as acute or chronic cholecystitis, choledocholithiasis, cholangitis, biliary pancreatitis, and rarely, gallbladder cancer^4,5^. For symptomatic patients and those who develop complications an emergency or elective cholecystectomy is usually indicated, making it one of the most frequently performed surgical procedures in the western world. Surgical cholecystectomy is not without physiological consequences and the discussion whether it is over- or under-used continues^4^. For one, a continuous secretion of bile into the duodenum replaces the physiological, pulsatile, meal-stimulated release from the gallbladder. This can lead to altered bowel motility^6^ paralleled by changes in the gut microbiota composition^7–9^. The latter have prompted speculations about a possible connection between certain microbial taxa and the risk of developing colorectal cancer, the incidence of which is increased after cholecystectomy^10^. On the other hand, changes in microbiota composition have been proposed to either increase or decrease the risk of developing gallstones—at least experimentally^11^. One disadvantage of previous studies was that they were mostly underpowered and focused on symptomatic gallstone carriers that were either scheduled for, or already subjected to cholecystectomy^7,8,12^. It therefore remains unresolved whether asymptomatic gallstone carriers are prone to intestinal microbial dysbiosis, whether certain gut microbial taxa reduce or increase the risk of developing gallstones (as suggested in rodent models^11^), or whether changes in abundance of certain gut microbial taxa increase the risk of gallstone carriers to become symptomatic and require cholecystectomy. To elucidate the role of the intestinal microbiome in gallstone disease, we investigated 1968 volunteers from the population-based Study-of-Health-in-Pomerania (SHIP) by 16S rRNA gene sequencing of fecal samples. We compared asymptomatic gallstone carriers with subjects after cholecystectomy and controls matched for potential confounding factors for microbiota composition.

## Results

We investigated the association of gallstone disease or cholecystectomy with intestinal microbiota profiles determined by 16S rRNA gene sequencing, comparing 580 individuals post-cholecystectomy (PCE, Table 1a) and 404 gallstone carriers (GC, Table 1b) to 580 and 404 control individuals without gallstone disease, respectively. All selected controls were matched to achieve similar distributions of the putative confounders age, sex, BMI, smoking, and diet (food frequency score, FFS) which are known to influence the gut microbiota composition^13–15^. None of the investigated participants received antibiotics at the time of sample collection.

**Table 1:** Phenotype characteristics of individuals post-cholecystectomy, gallstone carriers, and controls.

	PCE (n = 580)	Controls (n = 580)	p-value
**(a)**			
Age (years)	64.0 (55.0–72.0)	64.0 (55.0–71.0)	0.994
Female sex (%)	66.7	68.6	0.530
BMI (kg/m^2^)	29.7 (26.7–33.4)	29.6 (26.2–32.6)	0.460
Smoking (%)	13.6	13.8	1
FFS	15.0 (13.0–17.0)	15.0 (13.0–17.0)	0.825

	GC (n = 404)	Controls (n = 404)	p-value
**(b)**			
Age (years)	60.0 (51.0–69.0)	60.0 (50.0–68.0)	0.373
Female sex (%)	60.9%	62.1%	0.772
BMI (kg/m^2^)	29.0 (26.4–32.2)	29.2 (26.2–32.6)	0.749
Smoking (%)	20.3%	19.1%	0.723
FFS	15.0 (13.0–17.0)	14.0 (12.0–17.0)	0.171

Age, BMI and FFS are given as median (1st–3rd quartile). Female sex and smoking are stated as percentages. All numbers were rounded to one decimal place. *BMI* body mass index, *FFS* food frequency score, *GC* gallstone carriers, *PCE* individuals post-cholecystectomy, *n* number of cases. Statistical significance was assessed by Mann–Whitney test or Fisher's exact test for continuous or binary variables, respectively.

### Beta diversity analysis of individuals post-cholecystectomy and gallstone carriers as compared to controls

**Figure 1:**
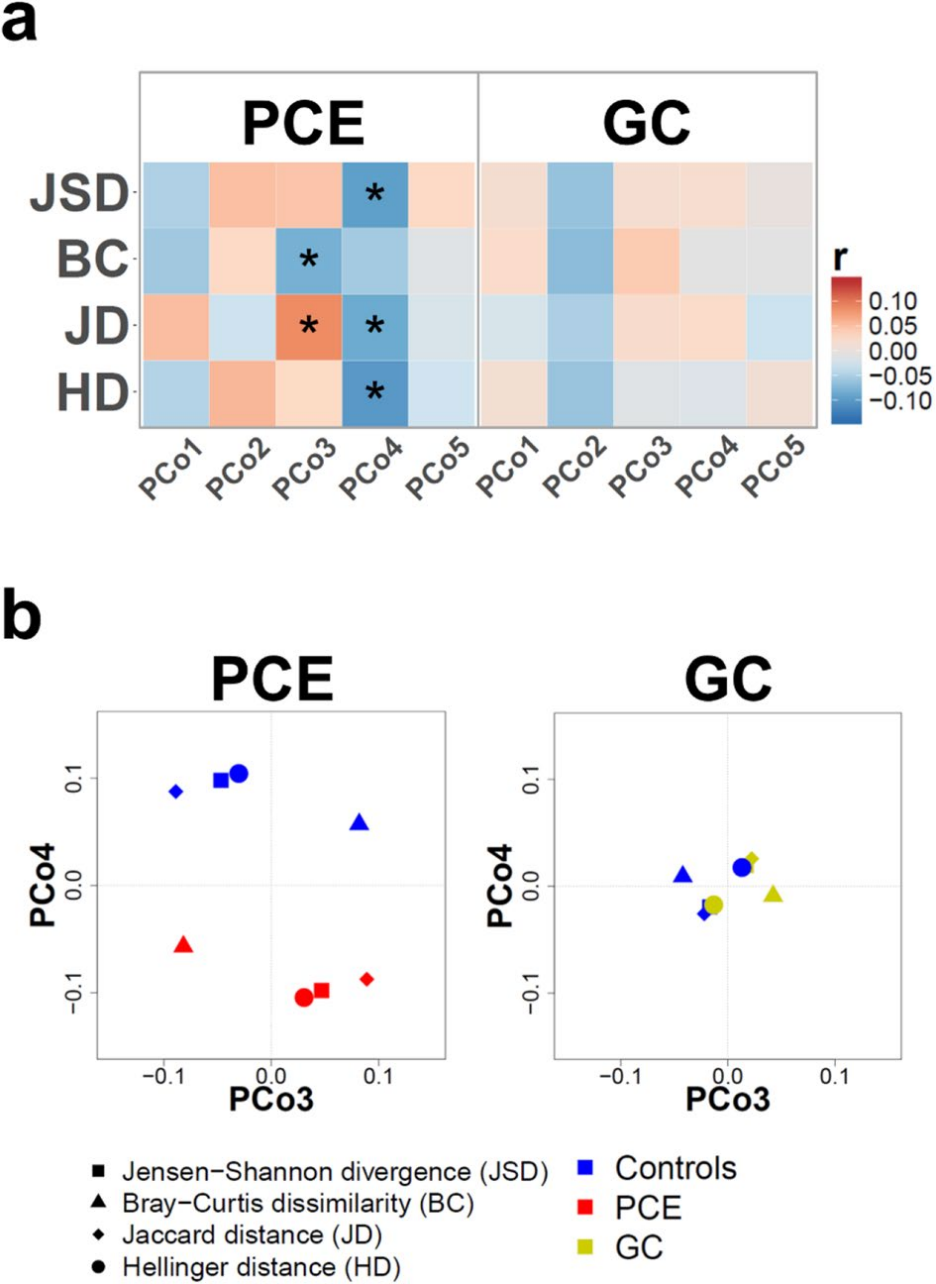
Cholecystectomy but not gallstone carrier status is associated with changes in beta diversity. (**a**) Heat map of point-biserial correlations between the group of individuals post-cholecystectomy (PCE, left) or gallstone carriers (GC, right) and beta diversity. The beta diversity indices Jensen-Shannon divergence (JSD), Bray–Curtis dissimilarity (BC), Jaccard distance (JD), and Hellinger distance (HD) were computed separately for PCE or GC cases and their respective control groups. The first five principle coordinates (PCo) are shown and significant correlations indicated by *. (**b**) Shown are the normalized PCo3 and PCo4 based on JSD, BC, JD, or HD for PCE (left) or GC (right). The centroids of PCE or GC cases are indicated by red or yellow symbols, respectively. The centroids of the respective control groups are displayed by blue symbols.

Beta diversity indices describe the variation between different samples, thereby estimating their similarity or dissimilarity, respectively. We calculated different beta diversity metrics, namely Jensen-Shannon divergence (JSD), Bray–Curtis dissimilarity (BC), Jaccard distance (JD), and Hellinger distance (HD). To investigate whether PCE or GC cases were associated with a shift in beta diversity compared to their respective control cohorts, we analyzed the correlation between these metrics and 'PCE' or 'GC' status. Figure 1a shows that PCE cases were associated with gut microbiota shifts mostly along principal coordinate (PCo) 3 and/or PCo4 of the different beta diversity indices. Figure 1b displays the shift of the centroids of PCE cases compared to controls. Further plots showing the individual sample distribution of PCE cases and controls score along PCo3 and PCo4 are given in Fig. S1. In contrast, GC cases did not exhibit any significant correlation with beta diversity as indicated in Fig. 1 and Fig. S2. These results were confirmed by permutational analysis of variance which indicated significant differences between the gut microbiota of PCE cases and controls based on JSD (p = 0.008), BC (p = 0.003), JD (p = 0.006), and HD (p = 0.004). GC cases, again, exhibited no significant differences compared to their controls in this analysis (Table S1).

### Taxon alterations at genus level comparing individuals post-cholecystectomy and gallstone carriers to controls

**Figure 2:**
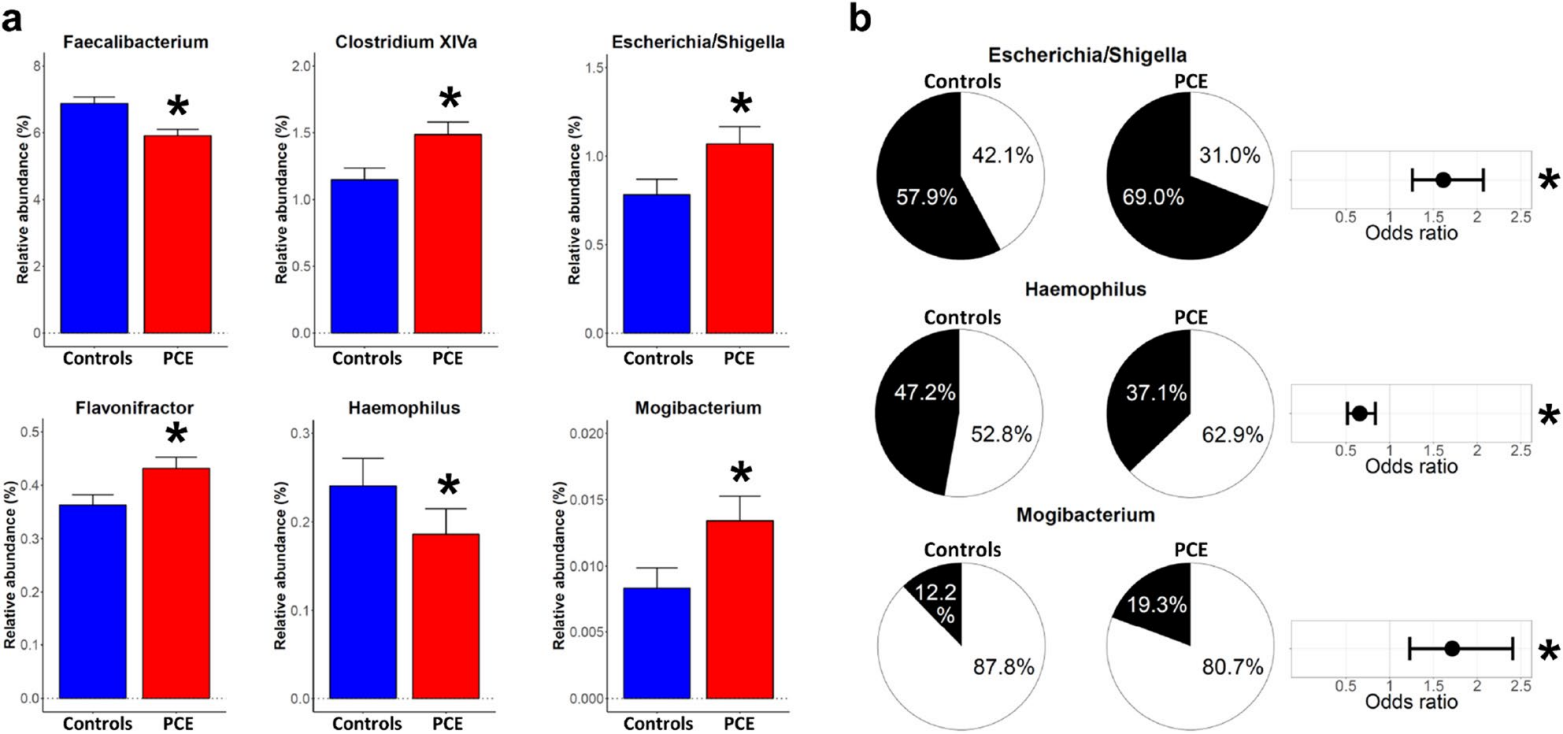
Differences in intestinal microbiota composition between individuals post-cholecystectomy (PCE) and controls without gallstone disease. (**a**) Barplots (mean + sem) depict all taxa at genus level with significantly (*) different abundance between controls (n = 580, blue) and PCE cases (n = 580, red). (**b**) Presence-absence alterations of gut microbiota. Pie charts are displaying the fraction of non-zero samples (in black). Shown are all taxa with significant increase or decrease in PCE compared to controls. The odds ratios represent the reduced or increased likelihood of the respective taxon to be present in PCE samples compared to controls.

The analyses of beta diversity suggested specific alterations in the gut microbiome of PCE cases. To identify these taxa, we compared continuous taxon abundance values between PCE cases and controls of all genera that were present in at least ten percent of all samples identifying numerous taxon associations (Table S2). Figure 2a shows all taxa with significant abundance changes in PCE cases including a decrease in *Faecalibacterium* (− 14.0%, q = 0.004) and *Haemophilus* (− 22.7%, q = 0.027) as well as an increase of *Clostridium XIVa* (29.5%, q = 0.021), *Escherichia/Shigella* (36.7%, q = 0.004), *Flavonifractor* (19.1%, q = 0.027), and *Mogibacterium* (60.7%, q = 0.021) when compared to controls.

The results of the beta diversity analysis implicated no large gut microbiota alterations in GC cases. To investigate, whether singular taxa express differences in their abundance between GC cases and controls, we performed a comparative analysis of continuous taxon abundance data as described above for PCE. After correction for multiple testing, no significant differences for any taxa were found (Table S3), confirming the negative global association results of the beta diversity analysis.

### Analysis of taxon presence-absence profiles of individuals post-cholecystectomy and gallstone carriers as compared to controls

Furthermore, we analyzed whether PCE cases exhibited changes in the mere presence or absence of specific genera (Fig. 2b and Table S4). This analysis focused again on all taxa that were present in at least ten percent of all samples. We found a positive association of PCE cases with the genera *Escherichia/Shigella* (q = 0.012) and *Mogibacterium* (q = 0.039). A negative association was identified with *Haemophilus* (q = 0.027). Again, if the same analysis was performed for GC cases, no significant differences were found compared to controls (Table S5).

### Comparison of predicted metagenomic profiles of individuals post-cholecystectomy and gallstone carriers as compared to controls

Metagenomic microbial profiles were predicted^16^ to evaluate functional differences of the gut microbiota. When comparing PCE cases and controls, we found a significant change in abundance of 59 predicted microbial pathways (Table S6), which belonged to the superclasses 'Degradation/Utilization/Assimilation' (n = 25), 'Biosynthesis' (n = 20), 'Generation of Precursor Metabolites and Energy' (n = 11) and others (n = 3). Corresponding to the increase in *Escherichia/Shigella*, predicted abundance of pathways for the biosynthesis of lipopolysaccharides (LPS) were increased by 35.7% in PCE cases (q = 0.032). No significant alterations in predicted microbial functions were found when comparing GC cases and controls (Table S7).

### Alpha diversity analysis of individuals post-cholecystectomy and gallstone carriers as compared to controls

**Figure 3:**
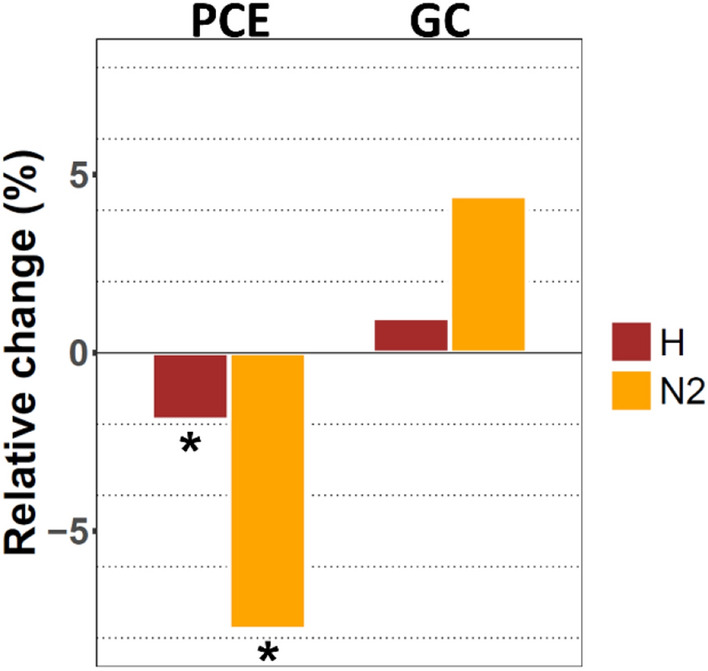
Changes in alpha diversity of intestinal microbiota in individuals post-cholecystectomy (PCE) and gallstone carriers (GC). Bars depict the relative change (%) of the mean for Shannon diversity index (H, brown), and Simpson diversity number (N2, orange) of PCE (n = 580) and GC cases (n = 404) compared to their respective control group (n = 580 and n = 404, respectively). *Indicates a significant result.

Alpha diversity metrics aim to describe the heterogeneity of microbial communities, the so-called 'within sample' diversity. To investigate the impact of gallstone disease on microbial diversity we calculated the alpha diversity indices 'Shannon diversity index' (H) and the 'Simpson diversity number' (N2). In PCE cases, median (1st–3rd quartile) scores for H and N2 were 4.4 (4.1–4.6), and 38.4 (26.2–53.0). Which were all lower than in controls with H 4.5 (4.2–4.7; p < 0.001) and N2 42.7 (29.7–56.3; p = 0.001). Comparing alpha diversity scores between GC cases and controls, however, did not exhibit any significant difference for the two alpha diversity metrics (Fig. 3).

### Direct gut microbiota comparison between individuals post-cholecystectomy and gallstone carriers

**Figure 4:**
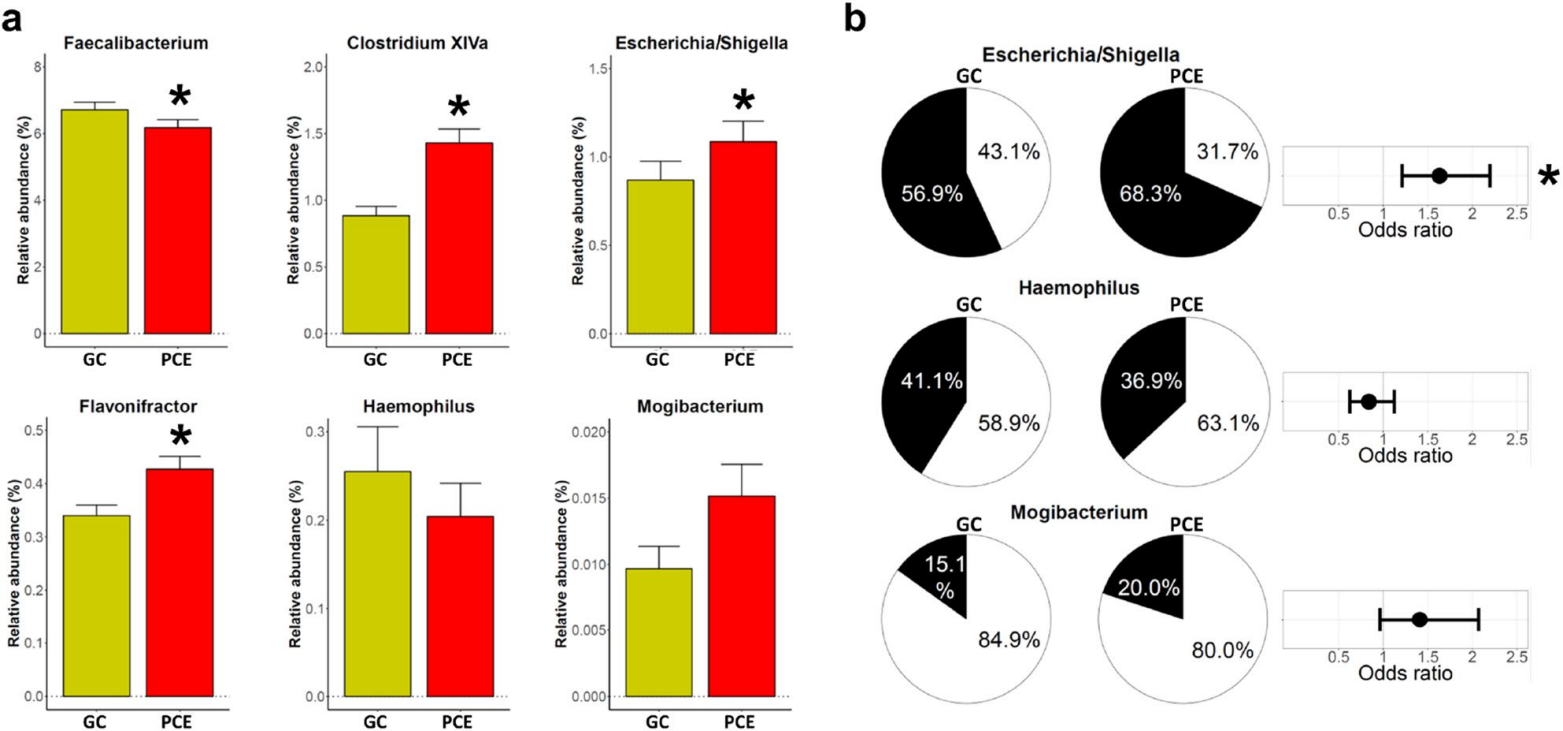
Differences in intestinal microbiota composition between individuals post-cholecystectomy (PCE) and gallstone carriers (GC). (**a**) Barplots (mean + sem) display taxon abundance of the PCE subsample (n = 404, red) and GC cases (n = 404, yellow). (**b**) Pie charts are displaying the fraction of non-zero samples (in black) of the PCE subsample and GC cases. The odds ratios represent the reduced or increased likelihood of the respective taxon to be present in the PCE subsample compared to GC cases. *Indicates a significant result.

Our analyses revealed numerous alterations in the gut microbiota of PCE cases when compared to controls without gallstone disease. We then investigated whether these specific gut microbiota alterations were also detectable when comparing PCE to GC cases. To this end, we selected a subsample of 404 PCE cases achieving a similar distribution of the putatively confounding factors age, sex, BMI, smoking and FFS as in the group of 404 GC cases (Table S8). When comparing these phenotypically matched PCE with GC cases, we could confirm lower levels of *Faecalibacterium* (p = 0.042) and higher levels of *Clostridium XIVa* (p < 0.001), *Escherichia*/*Shigella* (p < 0.001), and *Flavonifractor* (p = 0.002) in PCE cases (Fig. 4a). No significant differences were found in case of *Haemophilus* and *Mogibacterium*. When evaluating presence-absence data, we could confirm an increased proportion of samples with *Escherichia*/*Shigella* (p = 0.001) in PCE samples when compared to GC (Fig. 4b). Again, no significant differences were found for *Haemophilus* and *Mogibacterium*.

The median (1st–3rd quartile) alpha diversity scores H and N2 in this subsample of PCE cases were 4.4 (4.1–4.6) and 39.0 (27.8–52.8) and therefore lower than in GC cases with H 4.5 (4.2–4.7; p = 0.012), and N2 42.0 (30.5–55.6; p = 0.039). The median predicted genetic microbial potential for LPS biosynthesis was 0.8 (0.0–73.6) in the PCE subsample and 0.0 (0.0–39.2) in GC cases (p = 0.014).

## Discussion

Fecal microbiota profiles determined by 16S rRNA gene sequencing of individuals post-cholecystectomy (PCE), gallstone carriers (GC) and non-gallstone-disease controls were analyzed to characterize the gut microbiome changes associated with gallstone disease. We phenotypically matched the control cohorts to account for the known or putative confounding factors age^13^, sex, BMI, smoking, diet^14^, and sequencing batch and achieved a comparable distribution of parameters. In subjects with cholecystectomy we found numerous taxon abundance changes including an increase in abundance and presence of the genus *Escherichia*/*Shigella*. This confirms the observation of a smaller study which also identified an increased proportion of *Escherichia coli* in cholecystectomized individuals^8^. The Gram-negative facultative pathogenic bacterium *Escherichia coli* represents the most common isolate from either blood or bile in acute cholecystitis and/or cholangitis^17,18^. The detected overrepresentation of *Escherichia*/*Shigella* in the gut microbiome of PCE subjects may therefore increase the probability of developing such infections. Moreover, certain strains of *Escherichia coli* are known to induce DNA damage and increase gene mutation frequency. Increased levels of this taxon may therefore contribute to the known increase in risk for developing colorectal cancer in cholecystectomized individuals^8,10^. The predicted genetic microbial potential for LPS biosynthesis was also increased in PCE cases when compared to controls. LPS is a known promoting factor of tumorigenesis^19–21^ and LPS levels have been described to be increased in individuals suffering from colorectal cancer^22^. Therefore, increased LPS biosynthesis by Gram-negative bacteria after cholecystectomy may also promote the risk for colorectal cancer in these individuals. Another characteristic of the gut microbiota composition after cholecystectomy was a decrease of *Faecalibacterium*. This bacterium is an important producer of short-chain fatty acids, the main energy source of colonocytes^23^. It also exhibits anti-inflammatory features, partially through the production of anti-inflammatory proteins^24^. A reduction in *Faecalibacterium* has previously been reported in inflammatory bowel disease^25^. Its distinct reduction in cholecystectomized subjects may therefore represent a pro-inflammatory intestinal state. In contrast, another SCFA producer, *Clostridium XIVa*^26,27^ showed an increase in abundance. The magnitude of this increase was, however, much lower compared to the reduction of *Faecalibacterium* and may therefore not compensate for the depletion of the latter.

Analysis of the microbial alpha diversity scores revealed reduced levels after cholecystectomy in comparison to controls. In general, reduced alpha diversity scores have been associated with deteriorated health states such as obesity^28^, frailty^29^, or Crohn's disease^30^. In recurrent *Clostridioides difficile* associated diarrhea a reduced microbial diversity is believed to be the key feature for the persistence of the disease^31^. In the context of cholecystectomy, the reduced microbial diversity may result in an increased risk of microbiome perturbations and abdominal infections.

Based on a rodent model a connection between the development of gallstones and the gut microbiome has previously been proposed^11^. However, when we compared gallstone carriers with matched controls we found no significant difference in the gut microbiota composition—in contrast to the prominent changes found after cholecystectomy. The majority of gut microbiota changes identified after cholecystectomy in comparison to non-gallstone disease controls could be replicated when we compared post-cholecystectomy subjects with gallstone carriers. This indicates that interindividual variation in the intestinal microbiome is not likely to be a contributing factor to the development of gallstones in humans. However, this does not rule out that variation in the biliary microbiome may contribute to gallstone formation^32^. On the other hand, cholecystectomy is directly and prominently associated with intestinal microbiota dysbiosis.

The question remains why cholecystectomy induces such prominent changes in gut microbiota composition and diversity. One possible explanation would be a change in the dynamics of bile flow towards the small intestine. The altered availability of bile in the gut (continuous versus pulsatile) could favor species with different requirements regarding bile components, or bile-dependent break-down products of fat, for their metabolism. Alternatively, the altered intestinal motility after cholecystectomy^33^ could change the biophysical properties and fluid content in the colon, leading to more favorable conditions for certain taxa. Both explanations would be in line with the observation that the microbiome of the same patient changed in composition from the status before to that after cholecystectomy^7^. However, in that study no change in alpha diversity was detected after cholecystectomy, which is in contrast to our finding of a significantly reduced microbial diversity in PCE cases. An explanation for this difference could be small sample size of that study which included only 17 patients.

An alternative explanation for our findings would be that some of the, most likely adverse, changes, we identified in the gut microbiota of PCE subjects were already present before their respective cholecystectomy. In that case, the reduced gut microbial diversity as well as the increased abundance of *Escherichia*/*Shigella* could have contributed to turning an asymptomatic gallstone carrier into a symptomatic patient with infectious complications (such as cholecystitis or cholangitis), which ultimately prompted their cholecystectomy. A similar phenomenon has been reported in patients with non-Hodgkin lymphoma receiving chemotherapy, in whom a reduced microbial diversity prior to chemotherapy was associated with an increased rate of bloodstream infections during subsequent therapy^34^. Transmission of intestinal bacteria to the biliary tract may occur by ascending from the duodenum, via bloodstream or lymphatic vessels. Otherwise, although we excluded individuals under antibiotic treatment at the time of sample collection from the analysis, it is conceivable that a potential antibiotic treatment during the time of cholecystectomy may have caused lasting gut microbiota alterations in some of the PCE cases. Furthermore, the indication for cholecystectomy itself, which has not been recorded in this population-based study, may have affected the gut microbiota signatures.

The strength of this study includes its very large sample size and the thorough identification of individuals with gallstone disease or a status post-cholecystectomy by abdominal ultrasound. A potential drawback is the limitation that 16S rRNA gene sequencing does not allow us to identify the facultative pathogens on the species level.

This is currently the largest study investigating changes in fecal microbiota composition in subjects carrying asymptomatic gallstones or having undergone cholecystectomy for gallstone disease. While gallstone carriers showed no alterations in their gut microbiota compared to matched controls, we found individuals post-cholecystectomy to be characterized by a reduction in total microbial diversity and a depletion of the potentially beneficial genus *Faecalibacterium*, whereas the facultative pathogen *Escherichia*/*Shigella* was increased in abundance. While these significant changes were most likely caused by the altered flow of bile after cholecystectomy, we cannot rule out that they preexisted in affected subjects and contributed to the condition that ultimately required cholecystectomy. On the other hand, we found no evidence for the assumption that interindividual variation in the intestinal microbiota composition contributes to the risk of developing gallstones in the first place and, at the same time, carrying (asymptomatic) gallstones does not appear to induce changes in the gut microbiome. To what extent facultative pathogens with greater abundance after cholecystectomy contribute to post-cholecystectomy morbidity, specifically the increased colon cancer risk of cholecystectomized patients, can only be answered in large-scale long-term investigations. Whether or not the unfavorable microbiota composition after cholecystectomy can be restored to its original diversity by nutritional or microbiota-transfer techniques will have to be answered in randomized, controlled interventions. What we know at this point is that cholecystectomy comes at a cost, and with direct and unfavorable consequences for the intestinal microbiome. The results of the present study need to be considered in the discussion about a potential over- or under-use of cholecystectomy. They do not support the concept of performing cholecystectomy in asymptomatic gallstone carriers as this may result in microbiome perturbations with putatively adverse health effects for the human host.

## Methods

### Study participants

SHIP is a longitudinal population-based cohort study which aims to assess the incidence and prevalence of common risk factors and diseases^35^. It consists of the two independent cohorts SHIP (participants (n) = 4308, initial recruitment from 1997–2001 and recalled as SHIP-2 from 2008–2012) and SHIP-TREND (n = 4420, initial recruitment 2008–2012). Fecal samples for 16S rRNA gene sequencing were collected from SHIP-2 and SHIP-TREND participants. Abdominal ultrasound investigations were performed to evaluate the presence of gallstones or to confirm a status post-cholecystectomy. For the present work, a total of 6744 eligible datasets (SHIP-2, n = 2330; SHIP-TREND n = 4414) with diagnostic gallbladder ultrasound data were available, of which 148 participants were excluded due to indeterminate ultrasound gallbladder status. A further 159 individuals were excluded because of missing data regarding smoking, nutrition, or body mass index (BMI), which would have precluded phenotypic matching of cohorts. Another 89 datasets were removed due to the intake of antibiotics by the participants at the time of sample collection. The remaining datasets of 6348 individuals contained 687 subjects post-cholecystectomy and 491 cases with sonographically confirmed gallbladder stones. Of these, intestinal microbiota 16S rRNA gene sequencing data were informative for 580 post-cholecystectomy and 404 gallstone carrier subjects, which were included in the analysis. These cases were supplemented by an equal number of controls without gallstone disease with similar distributions of age, sex, BMI, smoking, diet, and sequencing batch. All participants provided written informed consent and the study was approved by the ethics committee of the University Medicine Greifswald (BB 39/08). All methods were carried out in accordance with the relevant guidelines and regulations (Declaration of Helsinki).

### 16S rRNA gene sequencing

16S rRNA gene sequencing of fecal samples was performed as described previously in detail^36^. In brief, stool samples were collected at home in a tube containing DNA stabilizing EDTA buffer and brought to the study center individually by the study participants or alternatively sent in by mail. DNA isolation was performed using the PSP Spin Stool DNA Kit (Stratec Biomedical AG, Birkenfeld, Germany). DNA isolates were then stored at − 20 °C until analysis. Amplification of the V1 and V2 regions of bacterial 16S rRNA genes was performed using the primer pair 27F and 338R and samples subsequently sequenced on a MiSeq platform (Illumina, San Diego, USA) using a dual-indexing approach.

### Assignment of taxonomy and predicted metagenomics

MiSeq FastQ files were created using CASAVA 1.8.2 (https://support.illumina.com/sequencing/sequencing_software/casava). The open-source software package DADA2^37^ (v.1.10) was used for amplicon-data processing which enables a single-nucleotide resolution of amplicons (amplicon sequence variants, ASVs). Data processing followed the authors' recommended procedure for large datasets (https://benjjneb.github.io/dada2/bigdata.html), adapted to the targeted V1-V2 amplicon. Briefly, on both reads, five bases were truncated from the 5′ end of the sequence. Forward and reverse reads were truncated to a length of 200 and 150 bp, respectively. A shorter resulting read length after truncation was possible if the sequence quality dropped below five. Read-pairs were discarded if they contained ambiguous bases, expected errors higher than two and when originating from PhiX spike-in. Error profiles were inferred using 1 million reads of the respective sequencing run, followed by dereplication, error correction and merging of forward and reverse reads. ASV abundance of tables of all samples were combined and chimeric amplicon sequences were identified and removed using the removeBimeraDenovo() function in consensus mode. Taxonomic annotation was assigned using a Bayesian classifier and the Ribosomal Database Project (RDP) training set version 16. Table S9 displays the read distribution after sequencing and several of the quality control steps. All data were rarefied to 10,000 reads per sample before computation of alpha or beta diversity or any between group comparisons at genus level. Prediction of metagenomic microbial functions was performed with the PICRUSt2 package^16^ using the standardized workflow as described at https://github.com/picrust/picrust2/wiki/Workflow with the ASVs from the DADA2 pipeline as input.

### Abdominal ultrasound

Abdominal ultrasound examinations were conducted by trained personnel. For assessment of the gallbladder status a high resolution device (Vivid i, GE Healthcare) was used in B mode. Possible outcomes were "no gallstones", "gallstones present", "post-cholecystectomy", or "indeterminate".

### Phenotypic data

BMI was calculated by dividing the body weight in kilogram by the square of the body height. For assessment of nutritional habits data from fifteen food categories were evaluated: meat (without sausage), sausage, fish, boiled potatoes, pasta, rice, raw vegetables, boiled vegetables, fruits, whole grain or black or crisp bread, oats and cornflakes, eggs, cake or cookies, sweets, and savory snacks. Frequency of food intake for each respective group was categorized as follows: "1" = daily or almost daily, "2" = several times per week, "3" = once a week, "4" = several times per month, "5" = once a month or less, "6" = never or almost never. Based on this data, a food frequency score (FFS) was calculated similar as described elsewhere^38,39^. In brief, the dietary patterns of the study subjects in each selected food group were classified according to the recommendations of the German Society of Nutrition to 0 (unfavorable), 1 (normal) or 2 (recommended) points (Table S10).

### Data and statistical analysis

For statistical analyses the statistical language 'R' (v.3.3.3, https://www.R-project.org/)^40^ was used. Plots were created with the package 'ggplot2’^41^. Matching of controls for PCE and GC cases was carried with the R package 'Matchit' using the option = ‘nearest’ which assigns controls by a distance-based approach^42^. During matching we accounted for age, sex, BMI, smoking, diet (FFS), and sequencing batch (Table S11). Computation of alpha diversity scores was performed using the R package 'vegan' ('Shannon diversity index', 'Simpson diversity number')^43^. For analysis of beta diversity the 'Jensen-Shannon Divergence’ (JSD) was determined as described elsewhere (http://enterotype.embl.de/enterotypes.html). The indices 'Bray–Curtis dissimilarity' (BC), 'Jaccard distance' (JD), and 'Hellinger distance' (HD) were computed using the R package 'vegan' (function 'vegdist' for BC and JD, function 'decostand' for HD). The 'vegan' function 'adonis' was used to perform permutational analysis of variance (1000 permutations). Principal coordinate analysis (PCoA) was conducted based on the different beta diversity indices using the 'vegan' function 'cmdscale'. For association of the traits PCE or GC with the first five PCos, point biserial correlations were computed ('stats' function 'cor.test'). The two-sided Mann–Whitney test ('stats' function 'wilcox.test') was applied for assessment of statistical significance in case of continuous phenotype, genus or predicted metagenomics data, whereas the two-sided Fisher's exact test ('stats' function 'fisher.test') was used for binary phenotype or genus data. When comparing genera or predicted metagenomic microbial pathways between PCE or GC cases and matched controls we only investigated those with a presence of at least ten percent in all samples. In case of genus or predicted metagenomics comparisons between PCE or GC cases and matched controls, p-values were adjusted for multiple testing by the method of Benjamini and Hochberg and thereafter called q-values. The same procedure was applied to p-values resulting from point biserial correlations between PCos and the traits PCE or GC. p- or q-values < 0.05 were considered significant.

## Supplementary Information

Supplementary Figures.

Supplementary Tables.

## Data Availability

All microbiome and phenotype data were obtained from the Study-of-Health-in-Pomerania (SHIP/SHIP-TREND) data management unit and can be applied for online through a data access application form (https://www.fvcm.med.uni-greifswald.de/dd_service/data_use_intro.php).
