# Supplementary material for: Carrying asymptomatic gallstones is not associated with changes in intestinal microbiota composition and diversity but cholecystectomy with significant dysbiosis
Source: Sci Rep. 2021 Mar 23;11:6677. doi: 10.1038/s41598-021-86247-6 (PMC7988160; doi:10.1038/s41598-021-86247-6)
Supplement: Supplementary file 1 — Supplementary Figures. [file 41598_2021_86247_MOESM1_ESM.pdf]

Carrying asymptomatic gallstones is not associated with changes in intestinal microbiota composition and diversity but cholecystectomy with significant dysbiosis

Fabian Frost[1]; Tim Kacprowski[2]; Malte Rühlemann[3]; Stefan Weiss[4], Corinna Bang[3]; Andre Franke[3]; Maik Pietzner[5]; Ali A Aghdassi[1]; Matthias Sendler[1], Uwe Völker[4]; Henry Völzke[6]; Julia Mayerle[1,7]; Frank U Weiss[1]; Georg Homuth[4] & Markus M Lerch[1]

Authors institutions:

1) Department of Medicine A, University Medicine Greifswald, Greifswald, Germany

2) Division Data Science in Biomedicine, Peter L. Reichertz Institute of TU Braunschweig and Medical School Hannover, Braunschweig, Germany

3) Institute of Clinical Molecular Biology, Christian Albrechts University of Kiel, Kiel, Germany

4) Department of Functional Genomics, Interfaculty Institute for Genetics and Functional Genomics, University Medicine Greifswald, Greifswald, Germany

5) Institute of Clinical Chemistry and Laboratory Medicine, University Medicine Greifswald, Greifswald, Germany

6) Institute for Community Medicine, University Medicine Greifswald, Greifswald, Germany

7) Department of Medicine II, University Hospital, LMU Munich, Germany

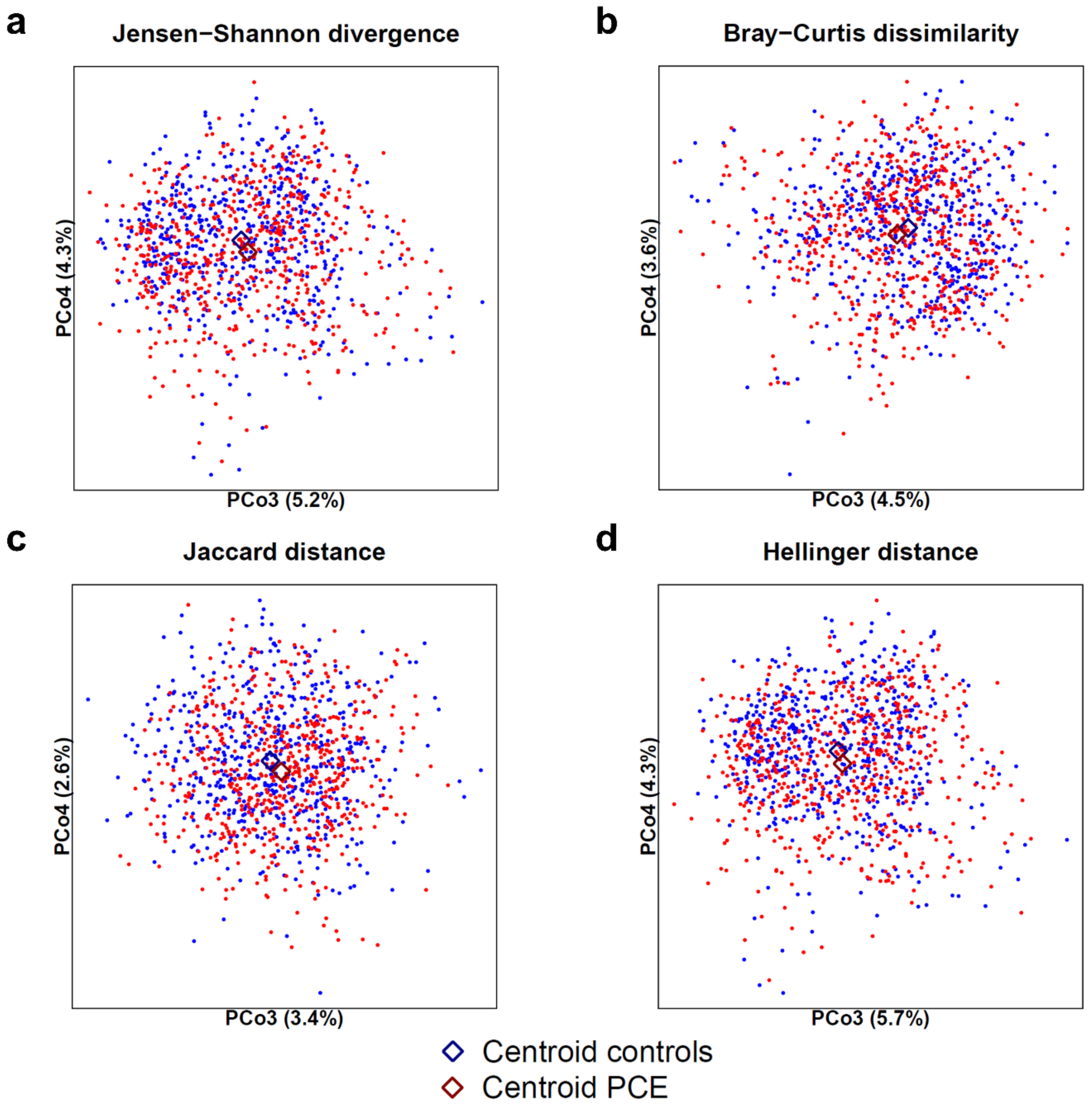

Fig. S1: Principal coordinate analysis (PCoA) of gut microbiota profiles from individuals post-cholecystectomy (PCE, n=580, red color) and matched controls (n=580, blue color). Shown are PCo3 and PCo4 of 1,160 gut microbiota samples. PCoA was performed based on **(a)** Jensen-Shannon divergence, **(b)** Bray-Curtis dissimilarity, **(c)** Jaccard distance, or **(d)** Hellinger distance.

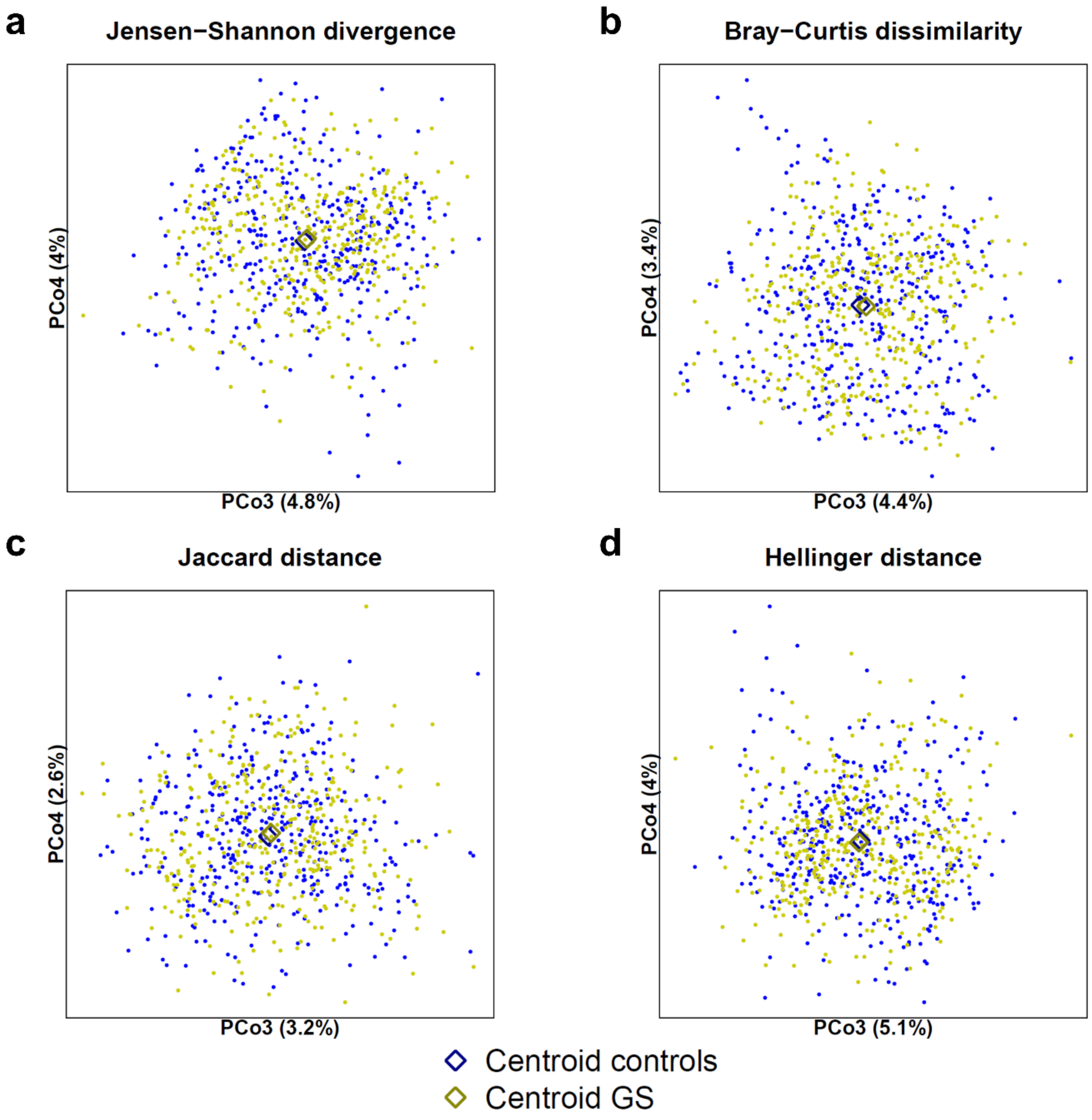

Fig. S2: Principal coordinate analysis (PCoA) of gut microbiota profiles from gallstone carriers (GC, n=404, yellow color) and matched controls (n=404, blue color). Shown are PCo3 and PCo4 of 808 gut microbiota samples. PCoA was performed based on **(a)** Jensen-Shannon divergence, **(b)** Bray-Curtis dissimilarity, **(c)** Jaccard distance, or **(d)** Hellinger distance.
